# Classification of estrogenic compounds by coupling high content analysis and machine learning algorithms

**DOI:** 10.1371/journal.pcbi.1008191

**Published:** 2020-09-24

**Authors:** Rajib Mukherjee, Burcu Beykal, Adam T. Szafran, Melis Onel, Fabio Stossi, Maureen G. Mancini, Dillon Lloyd, Fred A. Wright, Lan Zhou, Michael A. Mancini, Efstratios N. Pistikopoulos

**Affiliations:** 1 Texas A&M Energy Institute, Texas A&M University, College Station, TX, United States of America; 2 Artie McFerrin Department of Chemical Engineering, Texas A&M University, College Station, TX, United States of America; 3 Molecular and Cellular Biology, Baylor College of Medicine, Houston, TX, United States of America; 4 GCC Center for Advanced Microscopy and Image Informatics, Houston, TX, United States of America; 5 Bioinformatics Research Center, Center for Human Health and the Environment, Department of Statistics, North Carolina State University, Raleigh, NC, United States of America; 6 Department of Statistics, Texas A&M University, College Station, TX, United States of America; 7 Texas A&M University Institute for Bioscience and Technology, Houston, TX, United States of America; 8 Pharmacology and Chemical Genomics, Baylor College of Medicine, Houston, TX, United States of America; Institute for Disease Modeling, UNITED STATES

## Abstract

Environmental toxicants affect human health in various ways. Of the thousands of chemicals present in the environment, those with adverse effects on the endocrine system are referred to as endocrine-disrupting chemicals (EDCs). Here, we focused on a subclass of EDCs that impacts the estrogen receptor (ER), a pivotal transcriptional regulator in health and disease. Estrogenic activity of compounds can be measured by many *in vitro* or cell-based high throughput assays that record various endpoints from large pools of cells, and increasingly at the single-cell level. To simultaneously capture multiple mechanistic ER endpoints in individual cells that are affected by EDCs, we previously developed a sensitive high throughput/high content imaging assay that is based upon a stable cell line harboring a visible multicopy ER responsive transcription unit and expressing a green fluorescent protein (GFP) fusion of ER. High content analysis generates voluminous multiplex data comprised of minable features that describe numerous mechanistic endpoints. In this study, we present a machine learning pipeline for rapid, accurate, and sensitive assessment of the endocrine-disrupting potential of benchmark chemicals based on data generated from high content analysis. The multidimensional imaging data was used to train a classification model to ultimately predict the impact of unknown compounds on the ER, either as agonists or antagonists. To this end, both linear logistic regression and nonlinear Random Forest classifiers were benchmarked and evaluated for predicting the estrogenic activity of unknown compounds. Furthermore, through feature selection, data visualization, and model discrimination, the most informative features were identified for the classification of ER agonists/antagonists. The results of this data-driven study showed that highly accurate and generalized classification models with a minimum number of features can be constructed without loss of generality, where these machine learning models serve as a means for rapid mechanistic/phenotypic evaluation of the estrogenic potential of many chemicals.

## Author summary

Chemical contaminants or toxicants pose environmental and health-related risks for exposure. The ability to rapidly understand their biological impact, specifically on a key modulator of important physiological and pathological states in the human body is essential for diagnosing and avoiding undesirable health outcomes during environmental emergencies. In this study, we use advanced data analytics for creating statistical models that can accurately predict the endocrinological activity of toxic chemicals based on high throughput/high content image analysis data. We focus on a subclass of chemicals that affect the estrogen receptor (ER), which is a pivotal transcriptional regulator in health and disease. The multidimensional imaging data of these benchmark chemicals are used to train a classification model to ultimately predict the impact of unknown compounds on the ER, either as agonists or antagonists. To this end, we evaluate linear and nonlinear classifiers for predicting the estrogenic activity of unknown compounds and use feature selection, data visualization, and model discrimination methodologies to identify the most informative features for the classification of ER agonists/antagonists.

## Introduction

Characterization and prediction of the endocrine disruptive potential of complex chemical mixtures are essential to prevent their adverse effects on human health while understanding the biological pathways that lead to such undesirable health outcomes [1]. A key target of endocrine-disrupting chemicals (EDCs) is the Estrogen Receptor (ER), a modulator of important physiological and pathological states, including reproduction, metabolism, hormone-sensitive cancers, and obesity. There are many natural and man-made compounds that are capable of binding to the ER interfering with its activity, either as agonists, which activate a biological response (*i*.*e*., genistein, bisphenol A); or as antagonists, which generally compete with the endogenous hormones (*i*.*e*., 17β-Estradiol (E2)) to suppress the receptor function (*i*.*e*., 4-hydroxytamoxifen, fulvestrant). Mechanistically, E2 activates the ER pathway cascade through enabling a specific ER conformational change, receptor dimerization, DNA binding to regulatory elements in the genome, coregulator recruitment, and gene transcription activation/repression [2–4].

The estrogenic potential of different chemicals can be measured using cell-based or cell-free *in vitro* assays by recording several facets of the ER mechanism of action (*i*.*e*., ligand binding, cell proliferation, gene expression, *etc*.) [5–7]. Previously, a high content/high throughput microscopy-based assay in HeLa cells, engineered to harbor a visible multicopy integration of the ER response element present within the prolactin promoter/enhancer, was developed to capture several mechanistic steps of the ER pathway by imaging [5, 7–10]. Coupled with stable expression of GFP-ER⍺, this high content analysis-based approach allows the direct visualization of ligand-induced DNA binding by ER as a visually distinct nuclear spot/array when using standard fluorescent microscopy. Importantly, we observed differences in nuclear spot size and intensity when cells were treated with ER agonists and antagonists, which could potentially facilitate the characterization of ligands based upon their effect on ER activity when compared to known agonists and antagonists.

Furthermore, recent efforts have also focused on coupling high throughput experimentation with computational methods for enabling the rapid diagnosis of the estrogenic potential of various chemicals via *in silico* predictions [6, 11–16]. Judson *et al*. [6] used a linear model to predict the estrogenic activity of 1812 commercial and environmental chemicals based on the activity patterns across *in vitro* assays. The accuracy of this linear model is further tested by Browne *et al*. [13] for evaluating the ER agonist bioactivity, in which the authors postulated an integrated methodology to discriminate bioactivity from assay-specific interference. Similarly, Kleinstreuer *et al*. [15] used high throughput screening data of 1855 chemicals along with a linear additive model to predict the Androgen Receptor (AR) activity. Furthermore, Li and Gramatica [14] used AR data to develop quantitative structure-activity relationship (QSAR) models to classify binders as AR agonist or antagonist. The authors also investigated the performance of 4 different classification models, namely k-nearest neighbors (kNN), local lazy method (lazy IB1), alternating decision tree (ADTree), and an integrated consensus model [14]. In another study by Chierici *et al*. [16], deep learning and support vector machine (SVM) models were developed using the Collaborative Estrogen Receptor Activity Prediction Project (CERAPP) ToxCast data set for predicting the effects of EDCs on ER binding activity. A further detailed overview of *in* silico toxicity predictions using machine learning algorithms is provided in the notable review by Idakwo *et al*. [17].

Different from the aforementioned studies, we present here an integrated, data-driven framework for characterizing the endocrine-disrupting potential of chemicals that affect ER functions. In this framework, the high throughput/high content image analysis data, which provides hundreds of intensity and geometry-based features per cell, are used to generate classification models for promptly detecting the endocrine disruptive potential of unknown compounds as ER agonists or antagonists. We benchmark our approach using a group of control chemicals and present a systematic computational approach for predicting the estrogenic potential of unknown chemicals. Furthermore, by incorporating feature selection steps in this framework, we identify the most informative image-based features that enable a highly accurate separation between an ER agonist and antagonist without the loss of generality.

## Results and discussion

During and after environmental emergencies (*i*.*e*., hurricanes), humans are exposed to a number of chemicals, which in return creates an urgent need for the precise identification of their estrogenic potentials using rapid assessment techniques. Towards this goal, we collected 18 biologically independent experiments each containing 4 technical replicates of 45 benchmark compounds and 3 control treatments used at a single concentration with resulting high content analysis generating 7680 data points (192 observations x 40 features) per experiment. We aim to construct robust, generalized data-driven models that can accurately predict the endocrine-disrupting potential of unknown compounds from a limited number of experimental observations. To this end, one experimental data set is randomly selected for constructing the classification models among the 18 repeated image analysis experiments.

The selected data set is first pre-processed to extract the active subset of the 45 benchmark chemicals, and the uncorrelated features are identified using the computational methodology described in section *Materials and Methods*. A total of 64 observations were removed from each data set due to a failure to detect adequate visible DNA binding to characterize the compound. These observations were associated with compounds known to be inactive or very weak estrogens. Then, the clean data is split into training and test sets. Although it is common to split the data set using 80–20 or 70–30 rules (*i*.*e*., 80% training—20% testing), the experimental analysis on the 32 active benchmark chemicals yields an unbalanced data set due to the limited number antagonist versus agonist compounds. Hence, we constructed our training data set using all technical replicates of the 4 antagonist compounds and the 5 randomly selected agonist compounds with varying potency (Table 1) such that the classification models are trained on data where the distinct characteristics of the two classes of estrogenic activity are learned precisely. The remaining 23 agonist compounds and their respective 4 technical replicates in the chosen experimental data are reserved as the test set, enabling the fair assessment of the classification accuracy and other performance metrics of the model. As a result, the final training data matrix size with 9 chemicals becomes 36 observations x 5 features, the final testing data matrix size with 23 chemicals becomes 92 observations x 5 features of the selected image analysis experiment. The distribution of this training and testing data is also visualized through Principal Component Analysis (PCA) where a biplot of the first two components is provided in S1 Fig. Besides, the other 17 biologically independent image analysis experiments of the same list of benchmark chemicals are used for quantifying the classification model performance of estrogenic potential of chemicals subject to experimental noise.

**Table 1:** List of training compounds for the classification analysis. The agonist and antagonist compounds with varying ER potency selected for model training and their respective 4 technical replicates are included in the training data.

CASRN	Compound Name	ER Activity [6]	Potency [6]
115-32-2	Dicofol	Agonist	Very weak
56-53-1	Diethylstilbestrol	Agonist	Strong
53-16-7	Estrone	Agonist	Moderate
60168-88-9	Fenarimol	Agonist	Very weak
789-02-6	o,p'-DDT	Agonist	Weak
68392-35-8	4-Hydroxytamoxifen	Antagonist	-
82640-04-8	Raloxifene Hydrochloride	Antagonist	-
10540-29-1	Tamoxifen	Antagonist	-
54965-24-1	Tamoxifen citrate	Antagonist	-

### Linear classification results

The five biologically relevant features that were identified (see Materials and Methods for feature selection and descriptions) are used to construct individual linear classification models with a single descriptor. The best performing model is then selected out of these five logistic regression classifiers based on their Akaike Information Criteria (AIC) value, 5-fold training cross-validation (CV) accuracy, and testing accuracy. The results of linear classification training with the logistic regression model are provided in Table 2.

**Table 2:** Linear classification model training results with one experimental feature. The bootstrap confidence intervals (CI) for *β*_1_ are presented alongside with AIC, training CV accuracy, and testing accuracy results.

Experimental Feature in Model	*β*_1_	95% CI of *β*_1_	AIC	CV Accuracy	Testing Accuracy
Array to Nucleoplasm Intensity Ratio	7.12	(5.49, 7.59)	4.00	1.00	0.96
Array PI Variance	8.25	(4.48, 8.75)	4.00	1.00	0.87
Array Area	- 0.65	(-0.70, -0.59)	4.00	1.00	0.87
Array Mean PI	0.20	(0.12, 0.44)	27.92	0.87	0.85
Array Total PI	- 0.11	(-0.21, -0.03)	46.90	0.78	0.70

The results show that a logistic regression model with a single image analysis feature can accurately map the separation between agonist and antagonist compounds in the training phase. Specifically, we observe that linear classifiers trained with “Array PI Variance,” “Array to Nucleoplasm Intensity Ratio” and “Array Area” descriptors can classify the compounds with 100% training CV accuracy. The linear models with “Array Mean PI” and “Array Total PI” features have an inferior training performance, as the AIC values for these two models are higher and the CV accuracies are lower compared to the other models. This observation is also consistent with the Receiver Operating Characteristic (ROC) curves and the Area Under the Curve (AUC) values shown in S2 Fig.

Furthermore, the results show that “Array Area” and “Array Total PI” features have a negative effect on the linear classifier whereas the rest of the features have a positive effect. Specifically, values of the *β*_1_ parameter for “Array PI Variance” and “Array to Nucleoplasm Intensity Ratio” are the highest, respectively, indicating that a compound with higher values of these two features has an increased probability of being an antagonist. In addition, among these two most prominent features for the linear classification of estrogenic potentials of unknown chemicals, we observe that the model parameters of “Array to Nucleoplasm Intensity Ratio” and “Array PI Variance” have a relatively wider range of 95% confidence intervals.

Finally, the testing accuracy of trained models is evaluated using all technical replicates of the remaining 23 active compounds in this experiment. The testing accuracy results show that although “Array PI Variance” has a larger weight in the linear classifier compared to the rest of the descriptors, “Array to Nucleoplasm Intensity Ratio” has a higher testing accuracy for predicting the class information of the unseen chemicals. Table 2 shows that the linear classifier with “Array to Nucleoplasm Intensity Ratio” has a testing accuracy of 96% where this number drops to 87% when “Array PI Variance” is used as the sole predictor in the linear model. As a result, both predictors can perfectly map the separating linear boundary between the agonistic and antagonistic behaviors of chemicals in the training phase, whereas the linear model with “Array to Nucleoplasm Intensity Ratio” has a superior testing performance with a higher potential for achieving generality.

### Nonlinear classification results

The nonlinear classification analysis results are summarized in Table 3 where it shows the ranking of the experimental features based on the mean decrease in the Gini index score. The mean decrease in Gini index score is a measure of how strong a feature is for separating different classes of information, where prominent features lead to a larger decrease in this index. The results of the Random Forest (RF) model indicate that “Array to Nucleoplasm Intensity Ratio” is the top informative feature followed by “Array Area” and “Array PI Variance.” The mean decrease in Gini index for these 3 descriptors are very close to each other, showing that they are equally important for modeling the estrogenic potential of chemicals. The nonlinear classification results are consistent with the linear model, where these 3 features had 100% training CV accuracy and minimum AIC. Through careful consideration of the model parameters and the testing accuracy in linear models, we were able to distinguish “Array to Nucleoplasm Intensity Ratio” and “Array PI Variance” as the top two informative features for linear classification of agonist and antagonist compounds. Different than the linear analysis, we observe that the “Array Area” is the second most important feature for the nonlinear classification of estrogenic compounds whereas in the linear model the second-best feature was identified as “Array PI Variance.” Moreover, the model performance assessment with the training and testing data showed 100% and 93% classification accuracy, respectively. This high performance on the training data is expected as the model has learned the patterns within this set with high precision. The high testing accuracy of this model, on the other hand, shows that RF retains its predictive capability over a set of compounds that the model has not seen. As these initial tests show satisfactory results, further characterization of the model performance over biologically independent experiments is provided in the following section.

**Table 3:** Experimental features ranked with respect to their mean decrease in the Gini index.

Experimental Feature	Mean decrease in Gini index
Array to Nucleoplasm Intensity Ratio	5.79
Array Area	5.17
Array PI Variance	5.15
Array Mean PI	0.97
Array Total PI	0.19

### Visualization and evaluation of agonist and antagonist distributions

**Fig 1:**
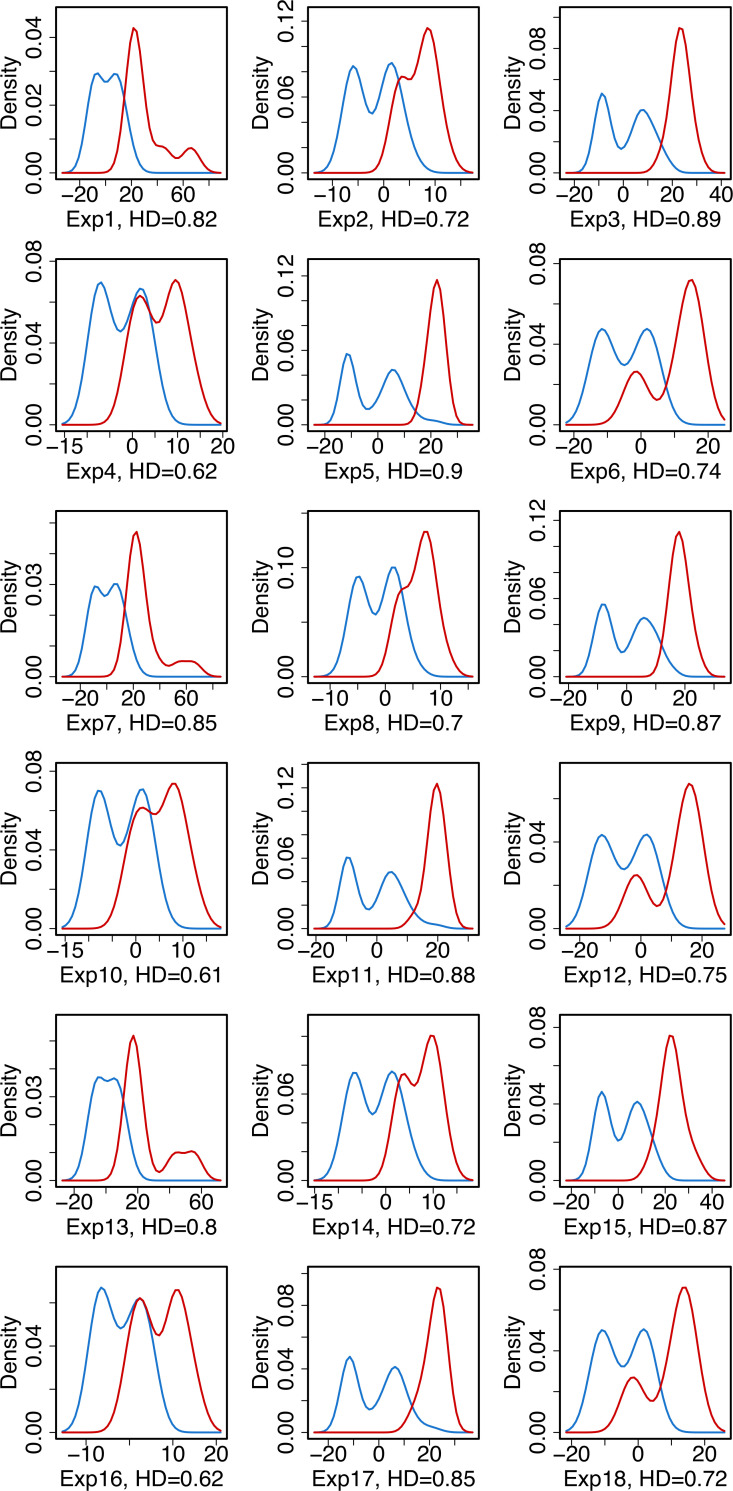
Density distribution of agonist (blue) and antagonist (red) compounds for the "Array to Nucleoplasm Intensity Ratio" feature.

**Fig 2:**
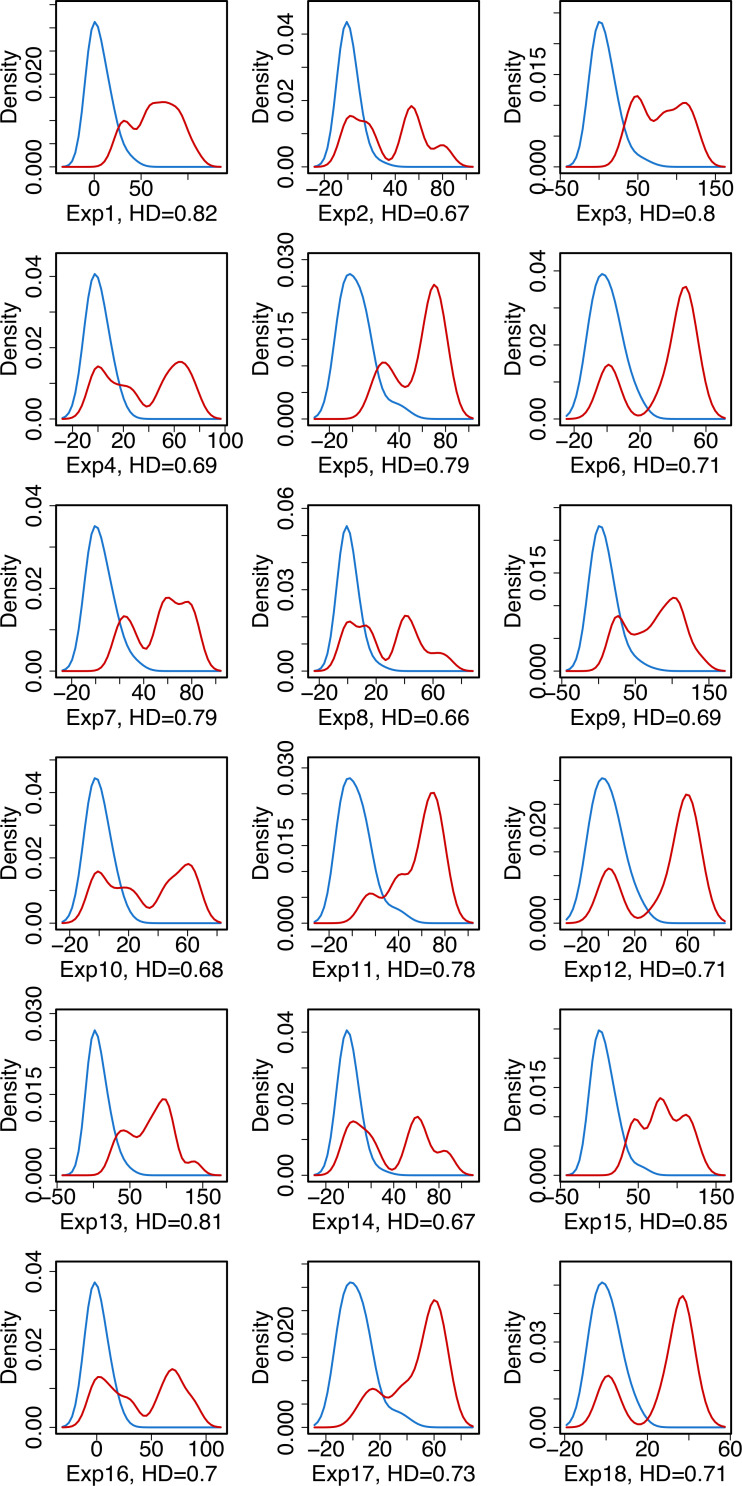
Density distribution of agonist (blue) and antagonist (red) compounds for the "Array PI Variance " feature.

In addition to the classification model development and using their mathematical properties to extract valuable information on the experimental features, additional insights on the separation between agonist and antagonist compounds are obtained through exploratory data analytics. To this end, we have plotted the density distributions of agonist/antagonist compounds for all independent experiments using the top important features identified by both linear and nonlinear classification analysis, namely the “Array to Nucleoplasm Intensity Ratio” and “Array PI Variance.” The density plots are provided in Figs 1 and 2 where the separation between agonistic and antagonistic behaviors of the chemicals, based on the values of the aforementioned descriptors, are visualized. The results in Figs 1 and 2 show that the “Array PI Variance” and “Array to Nucleoplasm Intensity Ratio” lead to a clear distinction between an agonist and antagonist for all experiments. To clearly distinguish between these two prominent features, the separation between the agonist and antagonist density distributions are quantified by calculating the Hellinger Distance (HD). This metric provides a measure of the distance between probability distributions and takes the values between 0 and 1, where smaller HD indicates that the two distributions are similar and the separation between them through the use of this feature is not statistically significant.

In Fig 1, the results show that the HD between the density distributions of agonist and antagonist compounds based on the “Array to Nucleoplasm Intensity Ratio” is high (min = 0.61, max = 0.90). Specifically, for independent experiments 3, 5, 9, 11, 15, and 17 there is a clear separation between agonistic and antagonistic behavior based on this descriptor, hence a linear classifier enables a highly accurate separation between these two estrogenic potential classes. However, in experiments 4, 10, and 16, a portion of the density distributions of these two estrogenic activities overlap and may lead to misclassification of compounds if the normalized “Array to Nucleoplasm Intensity Ratio” value of an agonist/antagonist falls into this overlapping region. Overall, we observe that ER antagonists possess strong signals for this experimental feature, thus enabling the separation of these two classes via a linear logistic regression model.

Similarly, in Fig 2, we observe that the HD distance of “Array PI Variance” measurements for all independent experiments is high (min = 0.66, max = 0.85), indicating that this feature is a valid descriptor for agonist versus antagonist separation. Different from Fig 1, here we observe that ER agonists have strong signals for this experimental feature in 12 out of 18 experiments. Furthermore, although the range of HD values are similar for both features, the number of experiments with HD > 0.8 is higher for “Array to Nucleoplasm Intensity Ratio” compared to “Array PI Variance.” This result indicates that “Array to Nucleoplasm Intensity Ratio” is more favorable for classification model building as this feature provides a clear distinction between ER agonists and antagonists over multiple biologically independent experiments.

Furthermore, we perform unpaired two-sample Wilcoxon test (*i*.*e*., Wilcoxon rank-sum test), a non-parametric alternative to the unpaired two-sample t-test, on the training data to compare the distributions of agonist and antagonist compounds per experimental feature. The Wilcoxon rank-sum test results in Table 4 show that the median measurements of agonist and antagonist compounds for “Array to Nucleoplasm Intensity Ratio,” “Array Area,” “Array PI Variance,” and “Array Mean PI” are different with p-values equal to zero. This statistically significant difference between agonist and antagonist distributions further strengthens the evidence that these features are appropriate for modeling the separation between two-classes of ER activity. In the case of “Array Total PI”, we fail to reject the null hypothesis, indicating that the distributions of agonist and antagonist compounds do not possess a statistically significant difference for this feature. Hence, the separation of the agonist and antagonist compounds with this descriptor is less desirable and will lead to misclassifications with worse predictive capability. This outcome is also consistent with linear and nonlinear classification results where this feature ranked the worst in training and testing accuracy (Table 2), as well as in feature importance (Table 3), respectively.

**Table 4:** P-values reported from the Wilcoxon rank-sum test. The bolded values represent p-value > 0.05.

Experimental Feature	p-value
Array Total PI	**0.067**
Array Area	0.000
Array Mean PI	0.000
Array PI Variance	0.000
Array to Nucleoplasm Intensity Ratio	0.000

### Classification model performance over independent experiments

**Fig 3:**
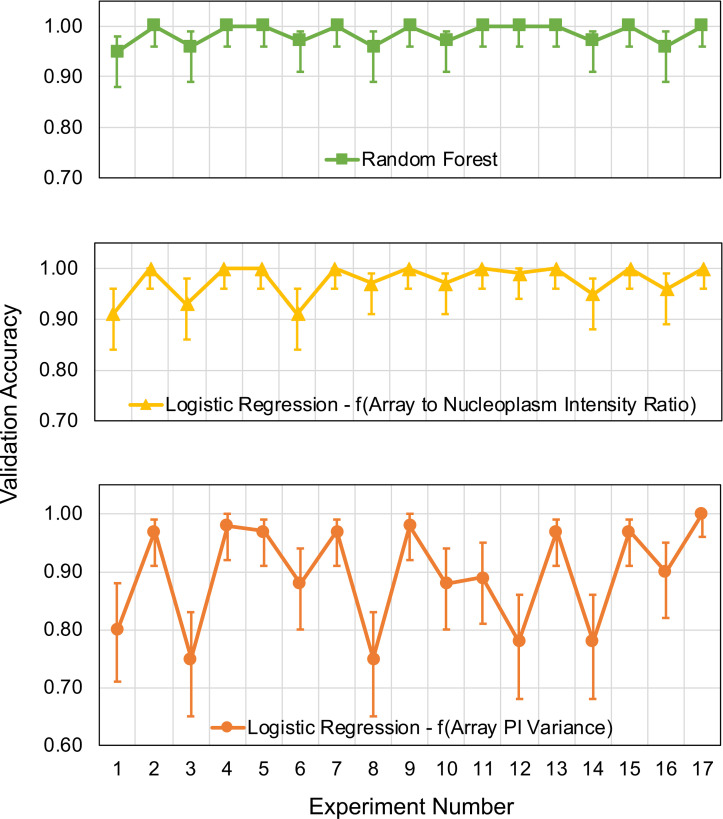
Model validation results with 23 unseen agonist compounds over 17 experiments for the logistic regression model as a function of “Array PI Variance,” the logistic regression model as a function of “Array to Nucleoplasm Intensity Ratio” and the Random Forest classifier.

In addition to the HD calculations and the Wilcoxon rank-sum test, the predictive capabilities of the trained and tested logistic regression (linear) and RF (nonlinear) classifiers are validated with a set of new 17 biologically independent experiments, and their corresponding predictive performance is quantified with a two-fold approach. First, the model performance is quantified using the 4 technical replicates of 23 unseen agonist compounds in 17 independent experiments that the model has not been trained on (Fig 3).

The blind validation accuracy results in Fig 3 shows that the predictive performance of the logistic regression model with “Array PI Variance” feature is inferior to the logistic regression model with “Array to Nucleoplasm Intensity Ratio” and the RF classifier. In 4 out of 17 experiments, the validation accuracy of this model is below 80% whereas, for the logistic regression model with “Array to Nucleoplasm Intensity Ratio” and the RF classifier, the validation accuracies exceed 90% for all experiments. Furthermore, the predictive capability of the latter two models is comparable to each other, where only in experiments 1 and 6, a relatively high difference between the validation accuracies of these two models is observed. In experiments 1 and 6, the validation accuracy of the RF model is 95% and 97%, respectively, whereas for the logistic regression model with “Array to Nucleoplasm Intensity Ratio” has a validation accuracy of 91% for both replicate testing. The lowest prediction accuracy for these two models is 95% for the RF classifier and 91% for the logistic regression model with “Array to Nucleoplasm Intensity Ratio” whereas this number drops to 75% for the logistic regression model with “Array PI Variance.” Moreover, the 95% CI of the validation accuracy is also provided in Fig 3. The RF and logistic regression models using “Array to Nucleoplasm Intensity Ratio” have tighter CI around the validation accuracy whereas the other logistic regression model has a wider CI on the prediction accuracy for all independent experiments. Overall, the blind validation accuracy results indicate that the nonlinear RF and linear logistic regression model with “Array to Nucleoplasm Intensity Ratio” are more favorable for predicting the estrogenic potential of unknown compounds as they have a more robust performance and can sustain their predictive capabilities over multiple biologically independent experiments.

**Fig 4:**
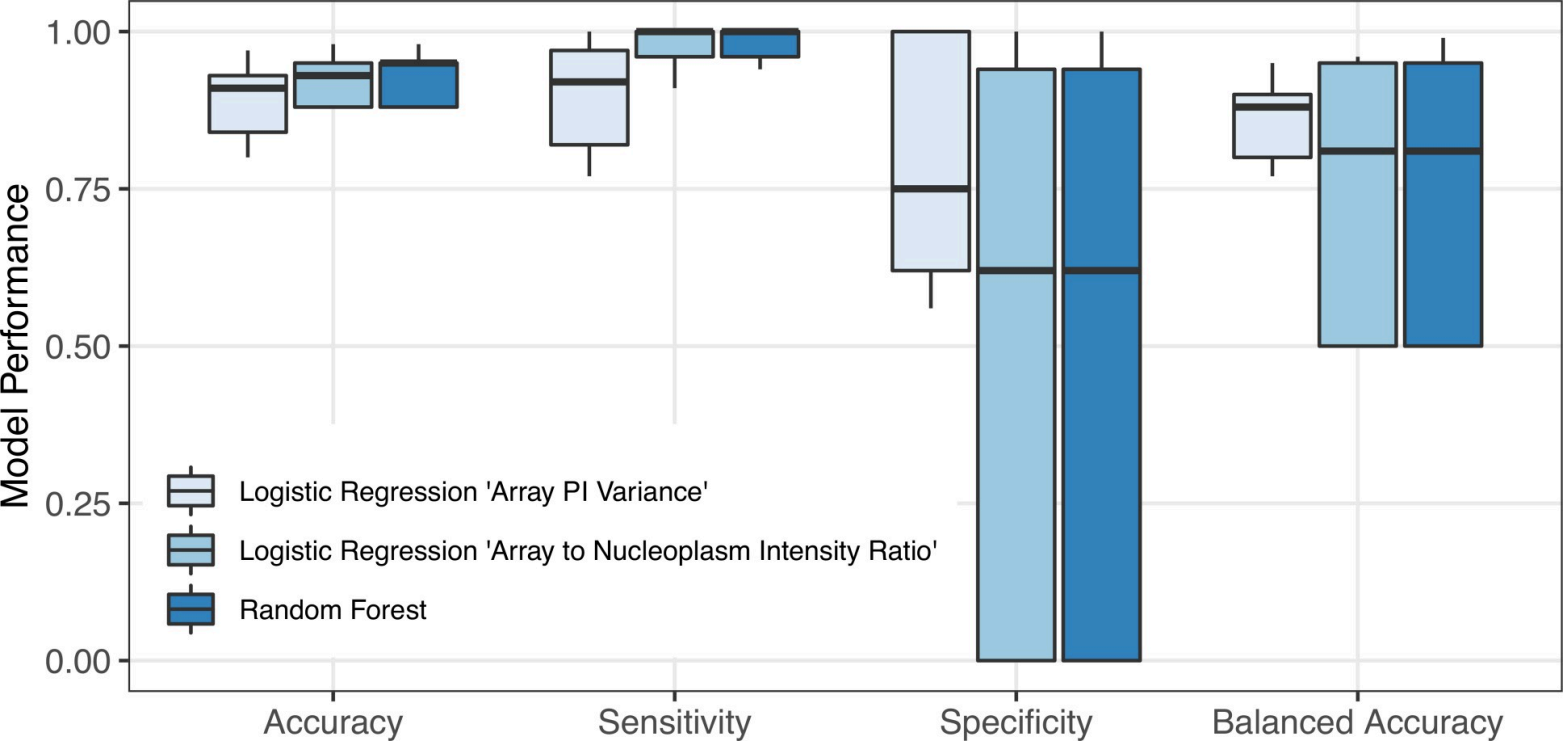
Effect of experimental variation on model performance. The 95% confidence intervals and the values used in constructing these box plots are provided in S1–S3 Tables.

Second, in Fig 4, accuracy, sensitivity, specificity, and balanced accuracy of different classification models are reported for these 17 experiments, but with all 32 active compounds. These results indicate that all three classification models have high accuracy and sensitivity for predicting the estrogenic potential of all active compounds considered in this study. Specifically, all models predict > 90% accuracy in 11 out of 17 experiments. Moreover, we observe that specificity and balanced accuracy of the logistic regression model with “Array PI Variance” is higher overall, when compared to other models, whereas the sensitivity of the RF classifier and logistic regression model with “Array to Nucleoplasm Intensity Ratio” is higher across different replicates. For the latter two models, we observe that the specificity value is zero for 5 experiments, indicating that these models identified all compounds as agonists and failed to classify the 4 antagonist compounds correctly. As a result, their balanced accuracy is also lower (50%), as this performance metric averages specificity and sensitivity values.

### Data quality monitoring

**Fig 5:**
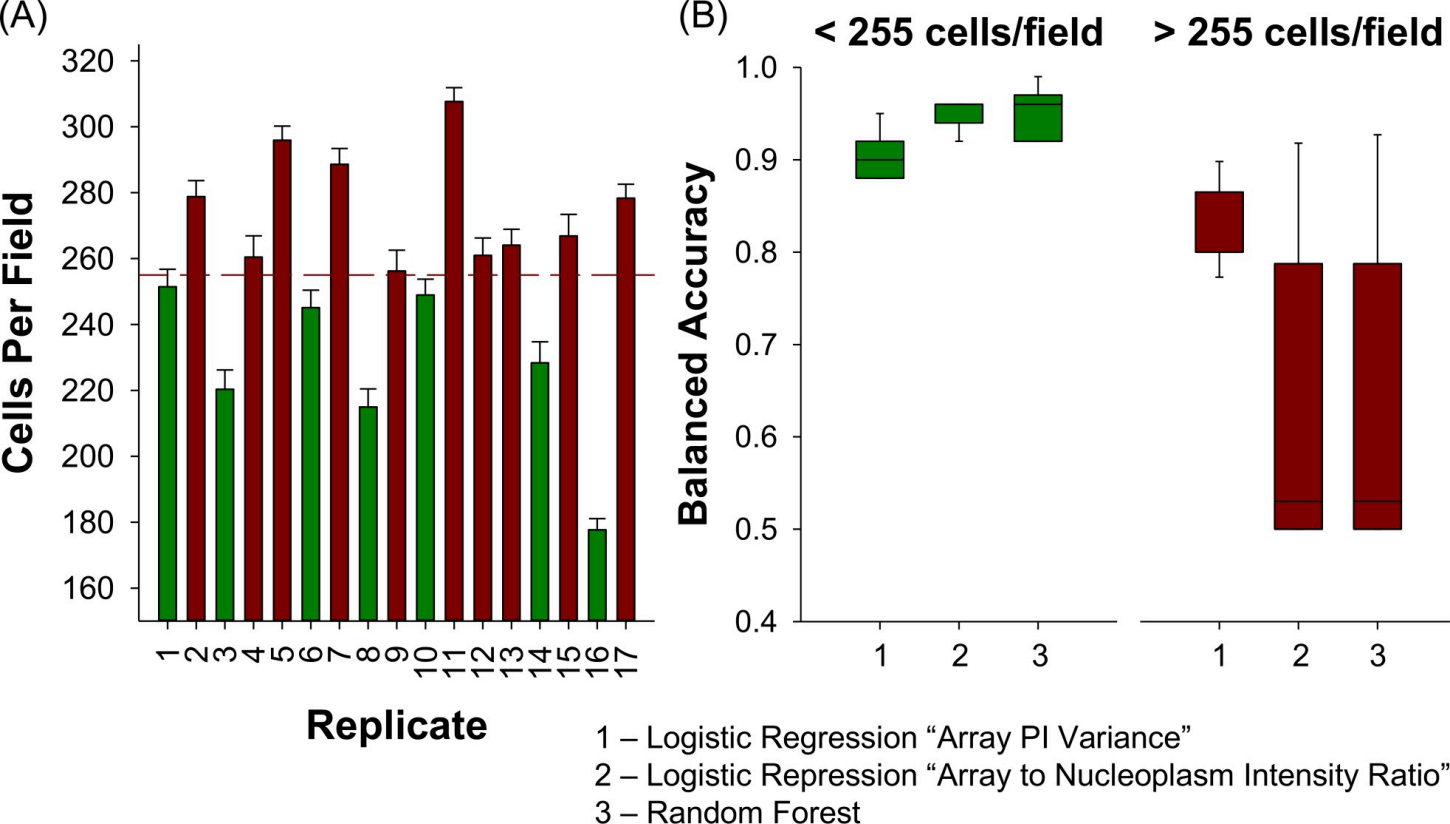
Cell density negatively affects model performance. (A) The average number of cells per microscopic field for independent experiments. The dashed line indicates the threshold for low- and high-density replicates. (B) Box plots of model balanced accuracy performance observed in low- and high-density experiments.

**Fig 6:**
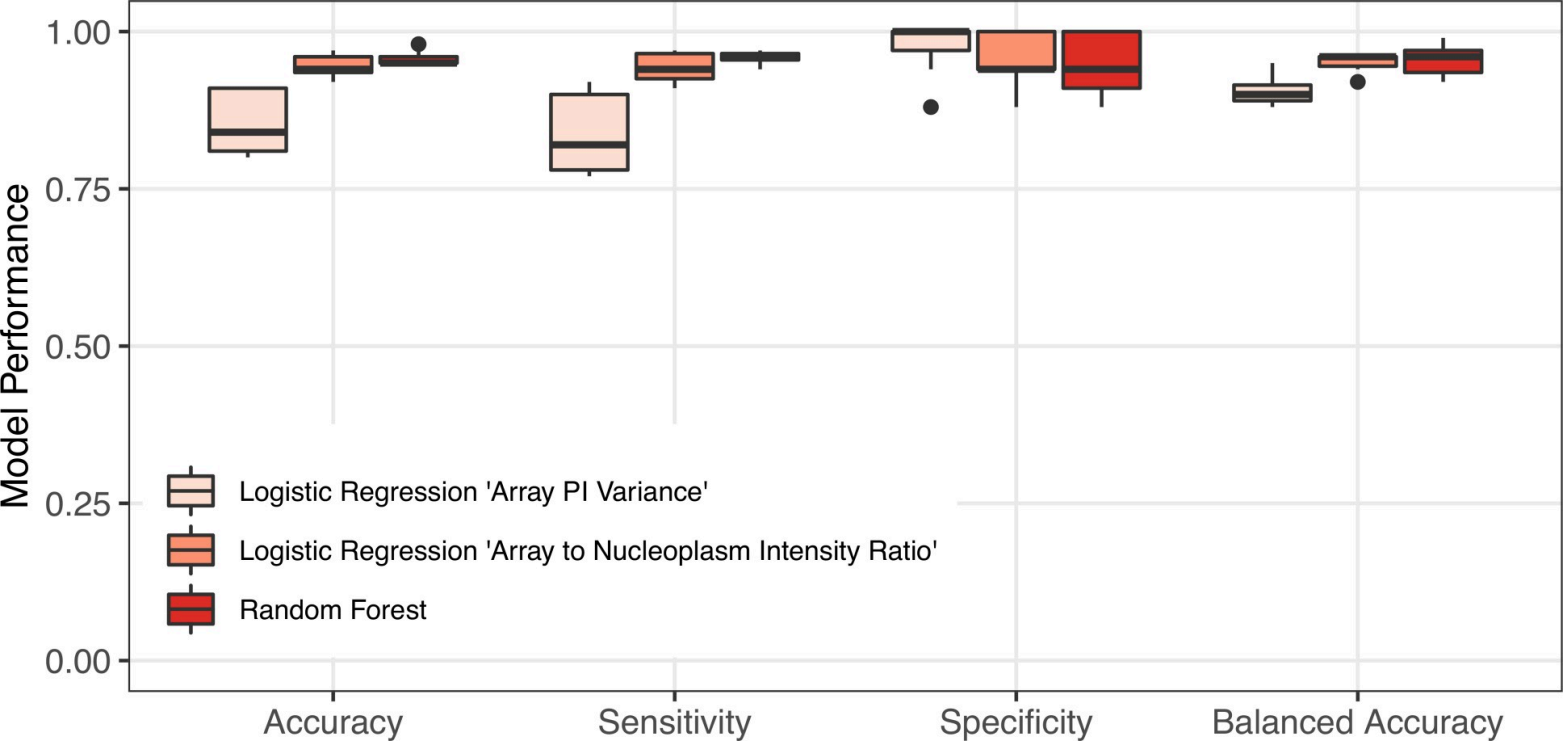
Data quality monitoring is essential for achieving high classification performance. Classification model performances with only low-density experiments.

This biphasic performance of each model on the identification of antagonist compounds suggests an underlying property of the experimental data sets that determines model performance. An exhaustive analysis of the correlation between data set features and model performance identified cell density (number of cells per microscopic image) as having a strong negative correlation (- 0.54 to -0.71) with model balanced accuracy. Cell density varies by 12.6% across independent experiments (Fig 5A). Using a threshold of 255 cells/well to divide them into ‘Low-Density’ and ‘High-Density’ experiments, we observe superior performance by all three models in the ‘Low-Density’ replicates (Fig 5B) with average balanced accuracy of 91% for the logistic regression model with “Array PI Variance” predictor and 95% for the other models, respectively. Reassessing the model performance metrics with only low-density experiments reveals that the RF and the logistic regression model with the “Array to Nucleoplasm Intensity Ratio” predictor are highly accurate and precise in predicting both classes of information (Fig 6). This result is not surprising based on the technical limitations of the assay and features used by the model. Increasing cell density increases the likelihood of any individual cell in a field being slightly out of focus. Since both “Array PI Variance” and “Array to Nucleoplasm Intensity Ratio” are contrast based features, they are dependent on focus quality. While the original optimization of cell density was based simply on the ability to detect the presence of a nuclear spot, slightly lower cell densities may be required to produce higher quality data required for high classification performance.

Overall, the nonlinear RF classification model and the linear logistic regression model with “Array to Nucleoplasm Intensity Ratio” are found to be highly accurate and robust for predicting the endocrine-disrupting potential of compounds on the ER. These holistic classification models allow us to predict the ER activity of compounds without running in separate agonist or antagonist modes and are shown to possess comparable classification performances with the previously reported values [6, 13, 16]. In addition, among 40 different experimental features studied in this work, “Array to Nucleoplasm Intensity Ratio” is found to be the top informative feature through a series of supervised and unsupervised analyses, and the results indicate that it is essential for predicting the ER activity of compounds through generalized predictive models.

As this study benchmarked the predictive capabilities of our hybrid data-driven classification models with satisfactory results, this methodology can be further used to predict the endocrine disruptive potential of novel chemicals. This prediction will require the high throughput high content image analysis experiment to be conducted that meets the data quality monitoring criteria (*i.e.*, low-density experiment), where a measurement for the 5 selected features will be recorded. For making predictions with the logistic regression model, we have already identified that this methodology provided superior predictions with the “Array to Nucleoplasm Intensity Ratio” feature. By using the model parameters that are identified as a result of this work and the pre-processed experimental measurement corresponding to “Array to Nucleoplasm Intensity Ratio”, the probability of this chemical being an antagonist is calculated by evaluating Eq 2 in *Materials and Methods*. If this probability is less than 0.5, the compound will be classified as an agonist, else as an antagonist. In the case of making predictions with the RF model, the pre-processed measurements of all 5 experimental features will be used to predict an outcome. As the RF is a decision tree-based model, an outcome will be calculated from all the trees and the majority vote is reported as the final class of the novel chemical. Hence, the established models can rapidly identify the endocrine disruptor potential of chemicals by function evaluation and subsequently classify them as ER agonists or antagonists. This prompt evaluation provides key information regarding the potential health effects of unknown chemicals and enables decision makers to take mitigating actions during exposures.

## Materials and methods

### Benchmark chemicals

Forty-five chemicals (Table 5) with varying estrogenic potentials were obtained from the United States Environmental Protection Agency (EPA) and were utilized for benchmarking our data-driven framework. The same compounds have been used by NIEHS/EPA as a set for developing computational models of the ER pathway [6].

**Table 5:** Summary of benchmark chemicals analyzed in this work. The ER activity information is adapted from Judson *et al*. [6].

CASRN	Compound Name	ER Activity [6]	Potency [6]
140-66-9	4-(1,1,3,3-Tetramethylbutyl)phenol	Agonist	Weak
599-64-4	4-Cumylphenol	Agonist	Weak
521-18-6	5α-Dihydrotestosterone	Agonist	Weak
57-91-0	17α-Estradiol	Agonist	Moderate
57-63-6	17α-Ethinyl estradiol	Agonist	Strong
58-18-4	17α-Methyltestosterone	Agonist	Very weak
50-28-2	17β-Estradiol	Agonist	Strong
520-36-5	Apigenin	Agonist	Very weak
85-68-7	Butylbenzyl phthalate	Agonist	Very weak
80-05-7	Bisphenol A	Agonist	Weak
77-40-7	Bisphenol B	Agonist	Weak
480-40-0	Chrysin	Agonist	Very weak
486-66-8	Daidzein	Agonist	Weak
117-81-7	Diethylhexyl phthalate	Agonist	Very weak
84-74-2	Di-n-butyl phthalate	Agonist	Very weak
115-32-2	Dicofol	Agonist	Very weak
56-53-1	Diethylstilbestrol	Agonist	Strong
53-16-7	Estrone	Agonist	Moderate
120-47-8	Ethylparaben	Agonist	Very weak
60168-88-9	Fenarimol	Agonist	Very weak
446-72-0	Genistein	Agonist	Weak
520-18-3	Kaempferol	Agonist	Very weak
143-50-0	Kepone	Agonist	Weak
84-16-2	meso-Hexestrol	Agonist	Strong
72-43-5	Methoxychlor	Agonist	Very weak
789-02-6	o,p'-DDT	Agonist	Weak
104-40-5	p-n-Nonylphenol	Agonist	Very weak
72-55-9	p,p'-DDE	Agonist	Very weak
68392-35-8	4-Hydroxytamoxifen	Antagonist	-
82640-04-8	Raloxifene Hydrochloride	Antagonist	-
10540-29-1	Tamoxifen	Antagonist	-
54965-24-1	Tamoxifen citrate	Antagonist	-
1912-24-9	Atrazine	Inactive	-
50-22-6	Corticosterone	Inactive	-
66-81-9	Cycloheximide	Inactive	-
13311-84-7	Flutamide	Inactive	-
52-86-8	Haloperidol	Inactive	-
52806-53-8	Hydroxyflutamide	Inactive	-
65277-42-1	Ketoconazole	Inactive	-
330-55-2	Linuron	Inactive	-
57-30-7	Phenobarbital sodium	Inactive	-
32809-16-8	Procymidone	Inactive	-
57-83-0	Progesterone	Inactive	-
50-55-5	Reserpine	Inactive	-
52-01-7	Spironolactone	Inactive	-

### Experimental data generation

**Fig 7:**
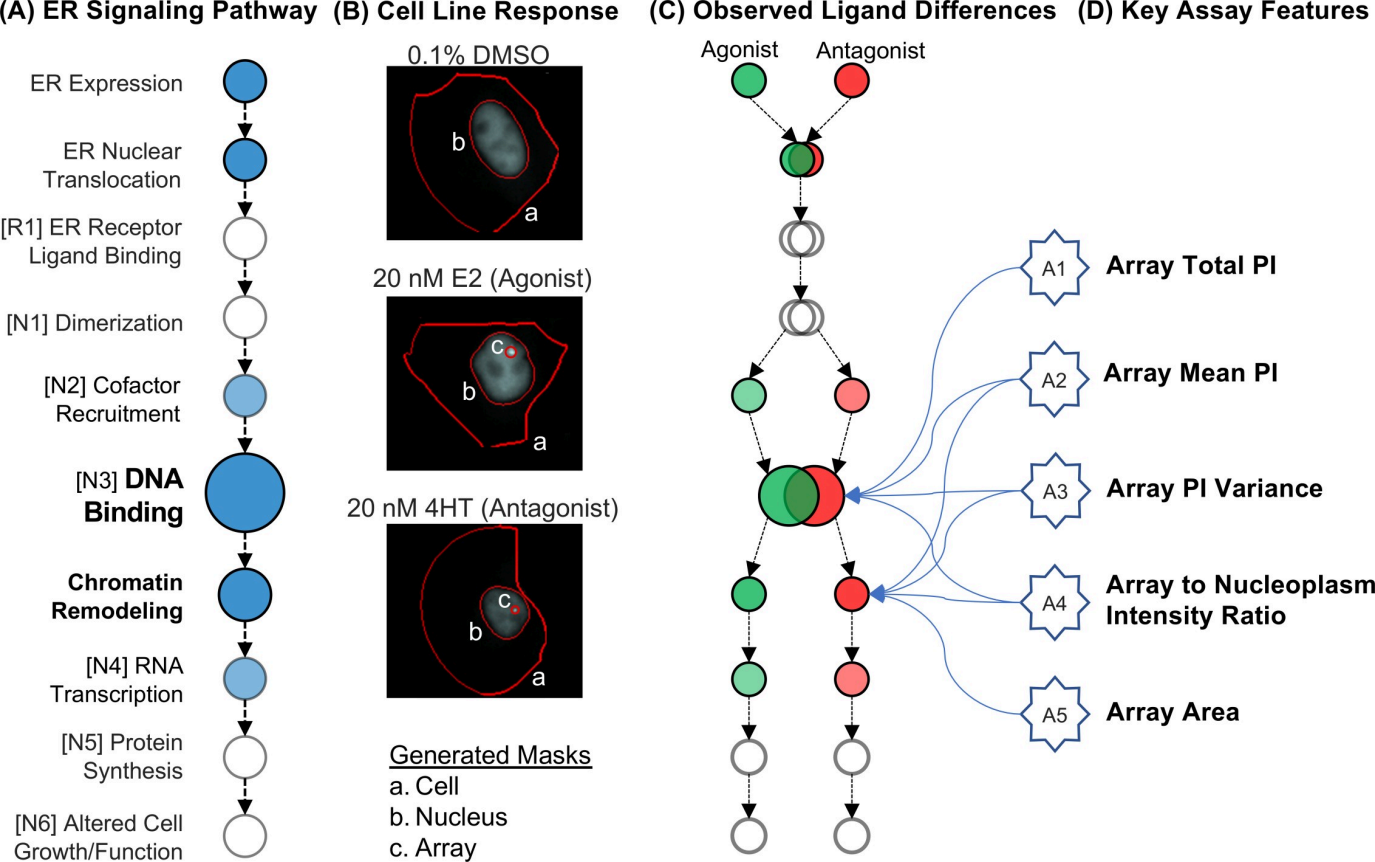
ER Signaling pathway endpoints detected using the GFP-ER⍺:PRL-HeLa cell model. (A) A graphical representation of the simplified ER genomic signaling pathway with nodes identified by Judson *et al*. indicated in brackets [6]. Colored circles indicate endpoints directly accessible using the GFP-ER⍺ alone and HCA. Shaded circles indicate endpoints that require additional labeling methods (antibody staining/mRNA FISH) to detect. The detection of chromatin binding is used to determine active/inactive samples in the current data sets. (B) Representative cell images from control samples. Red lines indicate the computational segmentation of the cell image. Regions identified are labeled with lower-case letters. (C) Graphical representation of the ER signaling pathway endpoints previously observed to be different when GFP-ER⍺:PRL-HeLa cells are treated with either agonists or antagonists [5, 7–10]. (D) Features selected for model development. Lines indicate ER pathway nodes measured by feature. Due to the interconnected nature of ER chromatin binding and chromatin remodeling, some features likely measure effects on multiple nodes.

High throughput microscopy and high content analysis-based experiments were performed using the GFP-ER⍺:PRL-HeLa cell line model following the experimental methodology described previously [5, 7–10]. This cell model allows for the direct simultaneous visualization of compound-dependent effects on several aspects of the ER signaling pathway, including ER expression, nuclear translocation, chromatin binding, and chromatin remodeling (Fig 7). Importantly, we have previously observed qualitative and quantitative differences in ER pathway signaling endpoints in the cell model-based compound activity class (Fig 7B and 7C) [5, 7–10]. 384 multiwell plates seeded with cells were treated for 2 hrs with a single concentration (10 uM) of 45 reference compounds provided by the EPA along with 3 control treatments. Control treatments included externally sourced agonist 17β-estradiol (E2, 20 nM), antagonist 4-hydroxytamoxifen (4HT, 20 nM), and negative control (DMSO, 0.5%). The experimental design included 4 technical replicates of each treatment, resulting in 192 different sample observations. Samples were imaged using an automated epifluorescent image cytometer and analyzed using myImageAnalysis/Pipeline Pilot to generate 40 different descriptors per cell [18]. The single-cell descriptors capture GFP-ERα fluorescence intensity (*i*.*e*., pixel intensity-PI) and morphology features of each cell, nucleus, and PRL array. The single-cell population is filtered to remove artifacts generated from cell toxicity, cell clusters, and incorrect segmentation. The remaining cell population (minimum of 500 cells per sample) data is averaged per sample to yield a data matrix size of 192 observations x 40 features, where the categorical output information for classification is provided in Table 5 in “ER Activity” column. A full list of experimental features is provided in [10].

### Computational methodology

**Fig 8:**
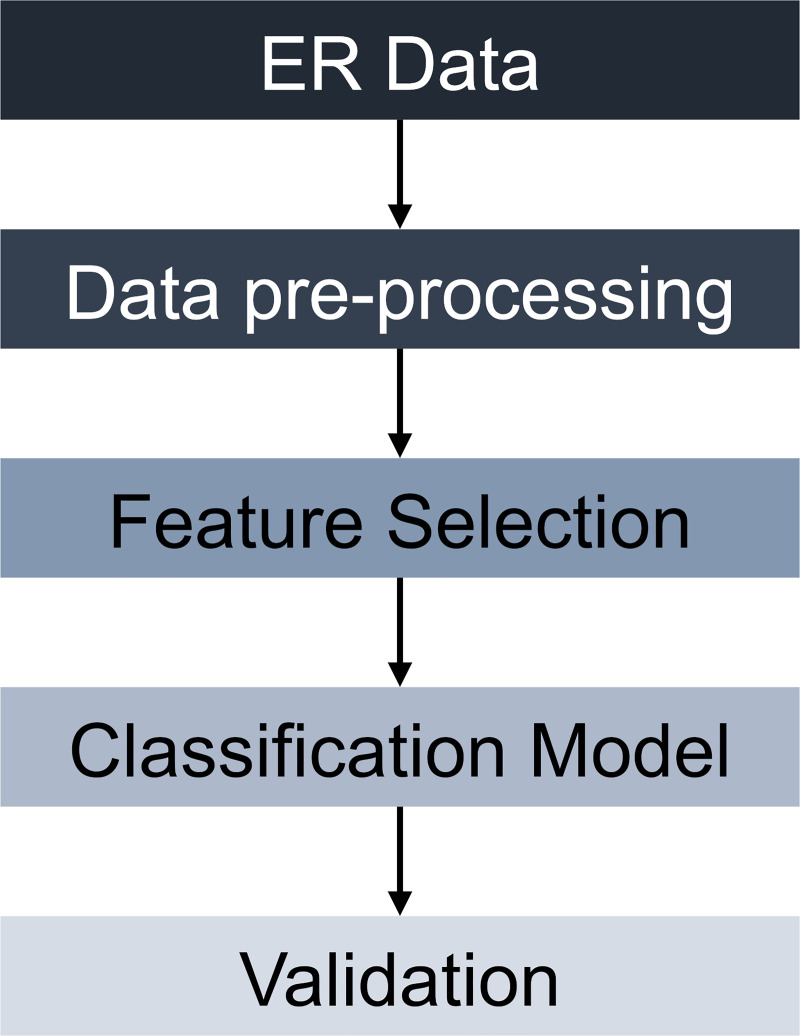
The classification framework for characterizing the estrogenic potential of chemicals. The computational methodology is documented via R Markdown and provided in S1 File.

The computational methodology follows a similar approach described in [19] where key steps of the framework are summarized in Fig 8. First, a series of pre-processing steps are executed to ensure accurate *in silico* predictions of ER activity with classification models. Once the pre-processing is completed, the data set is then passed on to the feature selection phase, where collinear features are eliminated from the analysis using hierarchical clustering. Later, a two-class classification problem is formulated using a subset of the features that are identified as independent and biologically relevant in the previous step. Finally, model validation is performed, and the predictive capability of the resulting classification model is quantified using model performance metrics. A detailed description of each step is provided below.

#### Data pre-processing

The pre-processing steps used in this analysis are: (1) missing data handling, (2) data cleaning, (3) outlier detection via unsupervised analysis, and (4) data normalization. The experimental data is first analyzed for missing data entries. If any missing data is detected, several procedures can be followed including, deletion of the entire row, deletion of the entire column, or data imputation [20]. As the experimental data from the image analysis did not have any missing points, no action is taken at this step, and the data matrix size of 192 observations x 40 features are retained.

**Fig 9:**
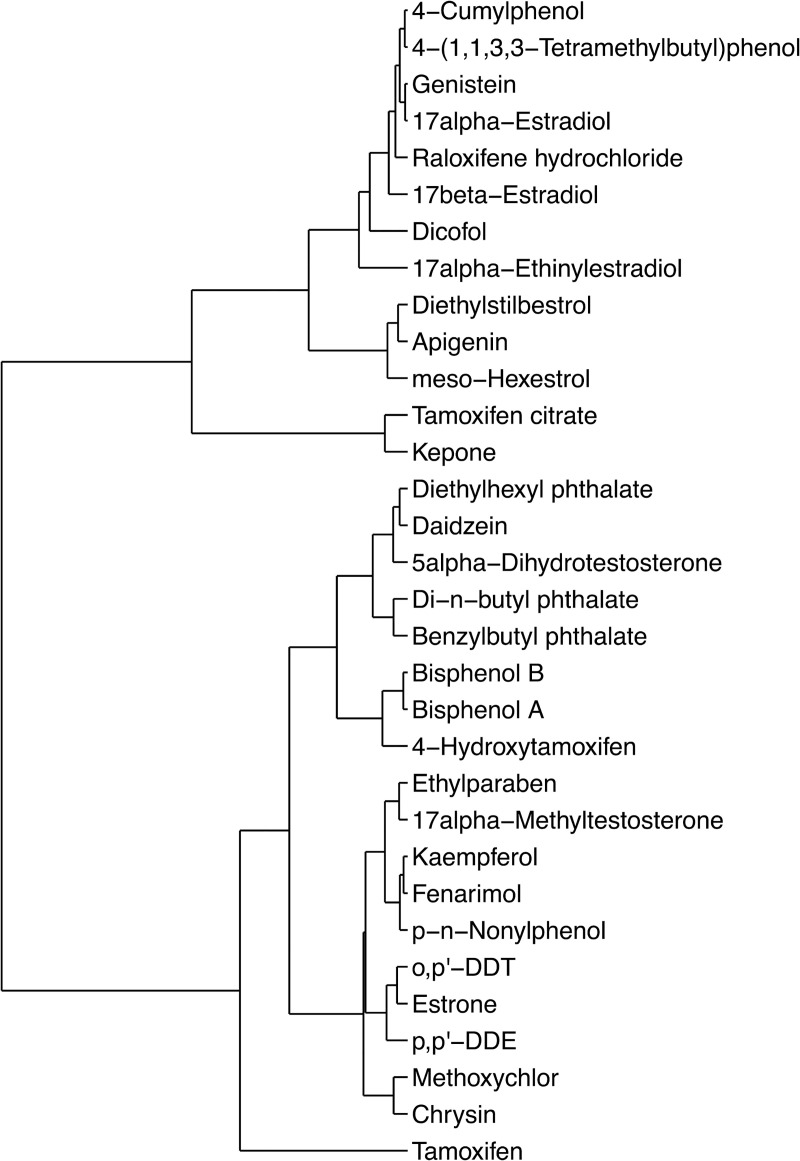
Outlier analysis via hierarchical clustering shown on a dendrogram tree.

In the next pre-processing step, the data set is cleaned by removing the observations corresponding to inactive compounds (Table 5). After this cleaning step, the data matrix size is reduced to 32 active compounds with 4 technical replicates per compound (128 observations) x 40 features. For outlier detection, the replicate observations of each compound are averaged, yielding a data matrix size of 32 average observations x 40 features. Hierarchical clustering is performed on the Euclidean distance-based dissimilarity matrix of this aggregate data with complete linkage. The clustering analysis is visualized using a dendrogram tree as shown in Fig 9. The results of the clustering analysis indicate that there are no global outliers present in the data set as none of the compounds significantly differ from each other. We observe that the active compounds are generally clustered under two groups based on their feature-specific patterns and are not presenting themselves on a separate branch at the root node of the dendrogram tree. As a result, the imaging data for all 32 compounds are viable for further analysis. The clustering is performed in R (version 4.0.0) using the hclust function under the stats library.

In the final pre-processing step, the remaining 32 active compounds are normalized using column-wise mean absolute deviation with respect to the control agonist E2 (Eq 1). The normalization is performed on the complete cleaned data set with technical replicates (Data matrix size: 128 observations x 40 features). In Eq 1, *i* represents the rows in the data set (*i*.*e*., technical replicates of each compound) and *j* represents the columns in the data set (*i*.*e*., features).

$${ER\;Data}_{i,j}^{normal}=\frac{{ER\;Data}_{i,j}^{original}-median({E2}_{j})}{mean({\left|{E2}_{i,j}-mean(E2_{j})\right|}_{j})}\forall i,j$$ (1)

#### Feature selection

The ultimate goal of this work is to present a data-driven methodology that integrates high content, high throughput image analysis-based data with machine learning algorithms for developing a robust, generalized classification model that accurately predicts the estrogenic potential of unknown chemicals. Within the scope of this work, as the image analysis provides numerous fluorescence intensity and morphology features, several challenges come to rise in classification model development: (1) Only a subset of experimental features may provide valuable knowledge for the separation of agonist/antagonist ER activity and identification of those is a challenging task; (2) A subset of the features may be highly correlated, and may cause bias, leading to loss of generality, precision and accuracy in the predictive capability of the data-driven model; (3) Modeling with a high number of features without an adequate amount of samples may lead to overfitting.

In this work, we address the aforementioned challenges by incorporating a feature selection step in our data-driven modeling framework. Feature selection or variable selection is one of the key processes in machine learning model building, where the aim is to identify a subset of features among many others that are uncorrelated and the most informative set of descriptors, for a given data-driven modeling problem. There is a growing interest within various fields of engineering and sciences for developing computationally efficient feature selection algorithms that enable the identification of the minimum number of features for maximum predictive capabilities in data-driven models [21–25]. Here, the feature selection is done in two steps: (1) Through hierarchical clustering for identifying the groups of similar and correlated features, and (2) Through a heuristic feature selection step, in which a single feature is selected from each cluster based on the ER pathway model presented in [6].

**Fig 10:**
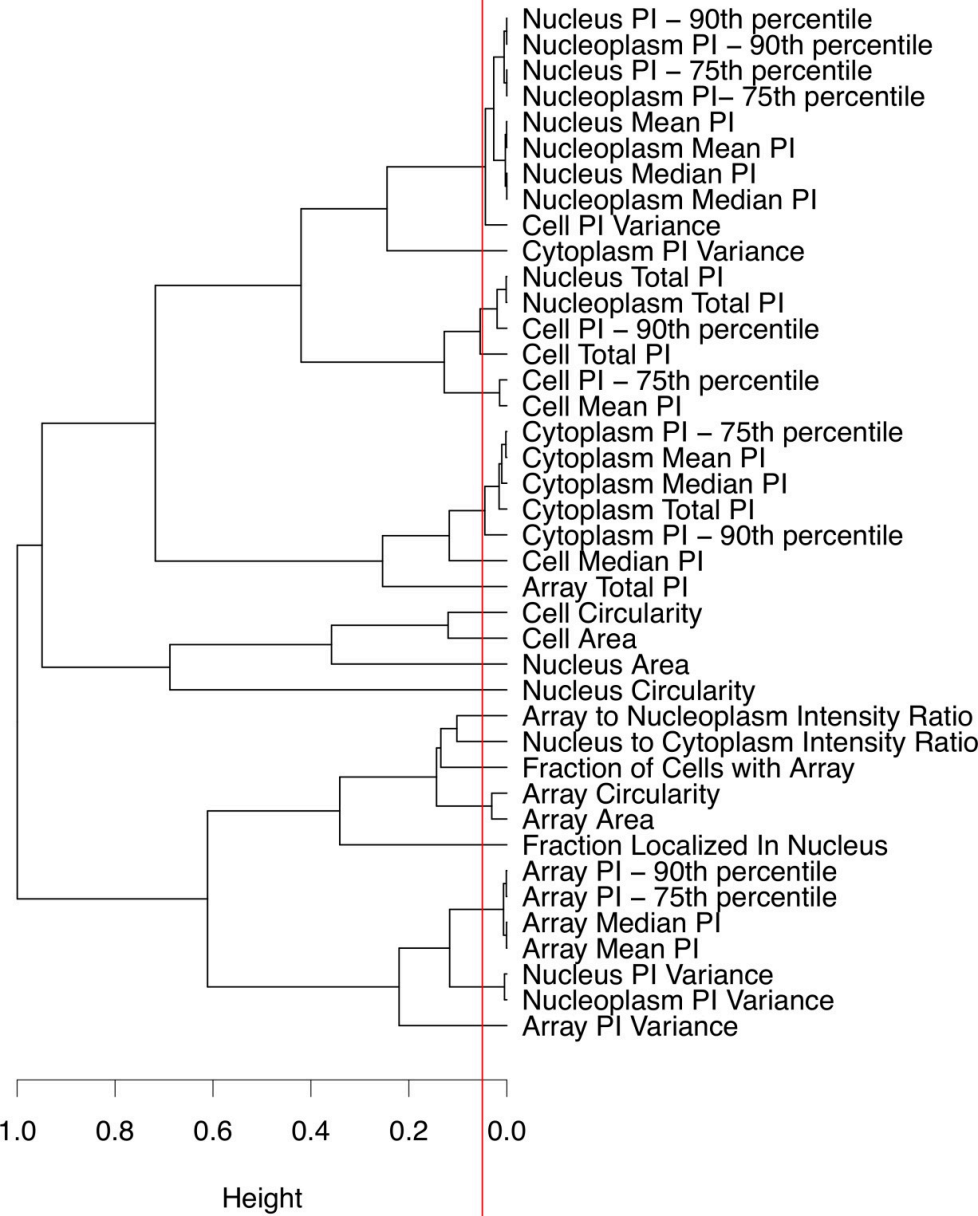
Uncorrelated feature selection using hierarchical clustering on pairwise feature similarity. The red line indicates the 5% similarity cutoff used for identifying independent feature groups.

In step 1, hierarchical clustering is performed on the pairwise similarity of experimental features, calculated using the Pearson correlation, with complete linkage. From the clusters of correlated features, groups that possess less than 5% similarity are identified as unique and uncorrelated for classification analysis. The clustering outcome is shown in Fig 10 with 20 independent feature groups of which we can select a subset of these for analysis. Like in the outlier analysis, the clustering for feature selection is performed in R (version 4.0.0) using the hclust function under the stats library.

In step 2, the goal is to further reduce the number of features for the classification analysis such that they are: (1) Selected from the independent groups of features following the clustering analysis (Fig 10); and, (2) the selected features are biologically relevant. The biological relevance of features is assessed through cross-referencing the image-based features to the ER pathway nodes presented in Judson *et al*. [6]. So, from 20 independent groups of features, the top 5 biologically relevant features (one shape and four PI-related descriptors) that are closely associated with a node on the ER signaling pathway, are selected. A summary of these features along with their descriptions are provided in Table 6 as well as in Fig 7D. This selection reduces the initial 40 image-derived features to 5, yielding a final data matrix size of 128 observations x 5 features that are passed on to the model development stage of the presented framework. The final data matrix is also visualized using PCA, where a biplot of the first two principal components is presented in S3 Fig.

**Table 6:** A subset of experimental features identified as uncorrelated and biologically significant for the classification analysis.

Feature Name	Image-Based Property of the Feature	ER Pathway Endpoint	Biological Relevance
Array Area	Size in pixels of visible promoter array	A5	Describes chromatin remodeling of promoter array
Array Mean PI	Average intensity of the GFP signal at the visible promoter array	A2	Describes level of GFP-ER⍺ binding to the promoter array
Array PI Variance	Statistical variance of GFP pixel intensity at the visible promoter array	A3	Describes GFP-ER⍺ intensity distribution at the visible promoter array
Array Total PI	Total intensity of the GFP signal at the visible promoter array	A1	Describes level of GFP-ER⍺ binding to the promoter array
Array to Nucleoplasm Intensity Ratio	Ratio of GFP intensity at visible promoter array to GFP intensity in the surrounding nucleoplasm	A4	Describes efficiency of GFP-ER⍺ binding the promoter array

### Classification model development using logistic regression and Random Forest classifier

Once the feature selection step is completed, the clean normalized data set is split into training and testing sets, and the training data are passed on to the model building phase for supervised analysis. In this work, the training set is a balanced subset of the active compounds that contain both agonist and antagonist chemicals and their corresponding 4 technical replicates, whereas the testing set is the remaining unseen active compounds with their corresponding 4 technical replicates, not used in the training phase.

Supervised learning algorithms are widely studied in many fields of engineering and sciences primarily in classification and regression-type problems for predicting either a categorical output or a continuous output, respectively [24, 26–32]. Classification is the problem of finding the categorical output of a new observation and distinguishing between different classes of information via statistical recognition of patterns in a training data set. In this study, we develop classification models to predict the endocrine disruptor activity of a set of benchmark chemicals. In this effort, both linear and nonlinear models are tested and their predictive performance on unknown chemicals is shown for comparison.

Linear classification is performed using the logistic regression model and the variables are selected using the Akaike Information Criterion (AIC). The logistic regression model with one predictor is provided in Eq 2,

$$P(antagonist)=\frac{1}{1+exp(-\beta_{o}-x\beta_{1})}$$ (2)

where *x* is the value of a predictor, *P*(*antagonist*) is the probability that the outcome is an “antagonist”, and *β*_1_ and *β_o_* are the parameters of the linear model where their values are estimated using the training data. The goal of the logistic regression training stage is two-fold: (1) To create a highly accurate and precise linear separating boundary between ER agonist and antagonist compounds; and, (2) to identify the most descriptive feature out of the 5 selected in the *Feature selection* step such that the *in silico* distinction between an ER agonist and antagonist is achieved without loss of generality. To this end, an exhaustive search is performed where individual logistic regression models, of the form presented in Eq 2, are constructed for all possible combinations of single features. The best performing model in this training phase with the minimum AIC, the highest CV training accuracy, and the highest testing accuracy is selected. In addition, the most informative feature for the linear classification problem is identified through analyzing the *β*_1_ parameter given that exp (−*β*_1_) quantifies the increase in the odds of a compound being an antagonist. As a result, an important feature with a larger weight in the closed-form equation will have a larger impact on the classification predictions.

For nonlinear classification, we use the RF algorithm with built-in feature ranking. RF is a nonparametric, tree-based ensemble learning method that uses multiple decision trees, independently constructed with a bootstrap sample of training data, to predict an outcome based on a majority vote [33]. The algorithm can identify “strong features” that causes a larger mean decrease in accuracy and display the relevance of features used in the training stage via the “Gini index” score [33, 34]. In our implementation, RF classifiers are constructed with 130 decision trees on the training data. The data-driven models for linear and nonlinear classification are implemented in R (version 4.0.0) using the glm function in stats library, and randomForest function in randomForest library, respectively.

#### Model validation and performance metrics

Model validation is done with a two-fold approach: (1) Using the technical replicates of unseen compounds that the model has not been trained on with model performance metrics quantified over independent experiments; and, (2) Using all technical replicates of active compounds in Table 5 with model performance metrics quantified over independent experiments. This two-fold approach allows us to quantify and observe the robustness of the unbiased predictive performance of the trained classification model over multiple biologically independent experiments and enable us to diagnose potential issues with the experimental data set (*i*.*e*., data quality monitoring).

The classification model performances are assessed using several evaluation metrics. These include accuracy, sensitivity, specificity, and balanced accuracy. Accuracy is defined as $\frac{TP+TN}{TP+FP+TN+FN}$, sensitivity (*i.e.*, true positive rate) is defined as $\frac{TP}{TP+FN}$, specificity is defined as $\frac{TN}{TN+FP}$, and the balanced accuracy is defined as the average of sensitivity and specificity, $\frac{1}{2}(\frac{TP}{TP+FN}+\frac{TN}{TN+FP})$. For this study, a “true positive” (TP) is defined as an agonist being correctly identified as an agonist and a “false positive” (FP) is defined as an agonist being misclassified as an antagonist. On the contrary, a “true negative” (TN) is defined as an antagonist being correctly classified as an antagonist and a “false negative” (FN) is defined as an antagonist being misclassified as an agonist.

## Conclusions

We have developed an integrated data-driven framework that enables the rapid identification of unknown pure chemicals that affect the estrogen receptor (ER) pathway as either agonists or antagonists. High throughput microscopy and high content analysis-based data are utilized to formulate highly accurate classification models by following a series of pre-processing, visualization, unsupervised, and supervised analysis steps. The framework is benchmarked with a set of chemicals with known ER activity. In the presented framework, a detailed pre-processing step is executed where: (1) experimental image analysis data is scanned for missing data points; (2) data is cleaned by removing the inactive compounds; (3) data set outliers are detected via hierarchical clustering; and, (4) experimental features are normalized via mean absolute deviation. Following pre-processing, the framework continues with a two-step feature selection methodology where uncorrelated features are first identified by hierarchical clustering using the pairwise similarity of the descriptors; secondly, the biologically relevant descriptor(s) are selected for analysis. Both linear and nonlinear classifiers are tested as a part of this framework for modeling endocrine-disrupting potentials of chemicals that affect ER functions, and their predictive performances are quantified via evaluation metrics. The linear and nonlinear classification model results show that high throughput microscopy and high content analysis-based experimental data can be used to train robust, highly accurate classifiers with a minimum number of features and sampling points (*i*.*e*., one feature for linear classification and five features for nonlinear classification). Through the results of this framework, we can identify the topmost important feature for the classification of ER agonists/antagonists without loss of generality and provide recommendations for the appropriate model selection. In addition, the presented data-driven framework serves as a guideline for rapidly scanning unknown chemicals and obtaining their estrogenic potentials with high accuracy. This property of the framework will be profound during environmental emergencies, where it is of the utmost importance to rapidly identify the potential biological risks of unknown chemicals.

## Supporting information

S1 File Access to experimental data files and analysis documentation via R Markdown. (DOCX)

S1 Fig Visualization of the technical replicates of training and testing compounds using Principal Component Analysis (PCA). The percentage of variance explained by each component is provided in the axis labels in parenthesis. The cumulative variance explained by these two components is 95.47%.
(DOCX)

S2 Fig The training receiver operating characteristic (ROC) curves and the area under the curve (AUC) values for the 5 predictors. The ROCs for the “Array Area,” “Array PI Variance,” and “Array to Nucleoplasm Intensity Ratio” perfectly overlap and represent a perfect classifier in the training phase with AUC = 1.
(DOCX)

S3 Fig Visualization of the technical replicates of agonist and antagonist compounds using Principal component analysis (PCA). The percentage of variance explained by each component is provided in the axis labels in parenthesis. The cumulative variance explained by these two components is 95.47%.
(DOCX)

S1 Table Logistic regression model validation results with all technical replicates of 32 active compounds for 17 biologically independent experiments with “Array PI Variance” as the model predictor. (DOCX)

S2 Table Logistic regression model validation results with all technical replicates of 32 active compounds for 17 biologically independent experiments with “Array to Nucleoplasm Intensity Ratio” as the model predictor. (DOCX)

S3 Table Random Forest model validation results with all technical replicates of 32 active compounds for 17 biologically independent experiments. (DOCX)

## References

[1] Dix DJ, Houck KA, Martin MT, Richard AM, Setzer RW, Kavlock RJ. The ToxCast program for prioritizing toxicity testing of environmental chemicals. Toxicological Sciences. 2006;95(1):5–12. 10.1093/toxsci/kfl103 16963515

[2] Nilsson S, Makela S, Treuter E, Tujague M, Thomsen J, Andersson Gr, et al Mechanisms of estrogen action. Physiological reviews. 2001;81(4):1535–65. 10.1152/physrev.2001.81.4.1535 11581496

[3] Hall JM, Couse JF, Korach KS. The multifaceted mechanisms of estradiol and estrogen receptor signaling. Journal of biological chemistry. 2001;276(40):36869–72. 10.1074/jbc.R100029200 11459850

[4] Hall JM, McDonnell DP. Coregulators in nuclear estrogen receptor action. Molecular interventions. 2005;5(6):343 10.1124/mi.5.6.7 16394250

[5] Szafran AT, Stossi F, Mancini MG, Walker CL, Mancini MA. Characterizing properties of non-estrogenic substituted bisphenol analogs using high throughput microscopy and image analysis. PloS one. 2017;12(7):e0180141 10.1371/journal.pone.0180141 28704378 PMC5509144

[6] Judson RS, Magpantay FM, Chickarmane V, Haskell C, Tania N, Taylor J, et al Integrated model of chemical perturbations of a biological pathway using 18 in vitro high-throughput screening assays for the estrogen receptor. Toxicological Sciences. 2015;148(1):137–54. 10.1093/toxsci/kfv168 26272952 PMC4635633

[7] Stossi F, Bolt MJ, Ashcroft FJ, Lamerdin JE, Melnick JS, Powell RT, et al Defining estrogenic mechanisms of bisphenol A analogs through high throughput microscopy-based contextual assays. Chemistry & biology. 2014;21(6):743–53. 10.1016/j.chembiol.2014.03.013 24856822 PMC4301571

[8] Sharp ZD, Mancini MG, Hinojos CA, Dai F, Berno V, Szafran AT, et al Estrogen-receptor-α exchange and chromatin dynamics are ligand-and domain-dependent. Journal of cell science. 2006;119(19):4101–16. 10.1242/jcs.03161 16968748

[9] Bolt M, Stossi F, Callison A, Mancini M, Dandekar R, Mancini M. Systems level-based RNAi screening by high content analysis identifies UBR5 as a regulator of estrogen receptor-α protein levels and activity. Oncogene. 2015;34(2):154 10.1038/onc.2013.550 24441042 PMC4871123

[10] Ashcroft F, Newberg J, Jones E, Mikic I, Mancini M. High content imaging-based assay to classify estrogen receptor-α ligands based on defined mechanistic outcomes. Gene. 2011;477(1–2):42–52. 10.1016/j.gene.2011.01.009 21256200 PMC3086628

[11] Martin T. Prediction of in vitro and in vivo oestrogen receptor activity using hierarchical clustering. SAR and QSAR in Environmental Research. 2016;27(1):17–30. 10.1080/1062936X.2015.1125945 26784454

[12] Chen Y, Cheng F, Sun L, Li W, Liu G, Tang Y. Computational models to predict endocrine-disrupting chemical binding with androgen or oestrogen receptors. Ecotoxicology and environmental safety. 2014;110:280–7. 10.1016/j.ecoenv.2014.08.026 25282305

[13] Browne P, Judson RS, Casey WM, Kleinstreuer NC, Thomas RS. Screening chemicals for estrogen receptor bioactivity using a computational model. Environmental science & technology. 2015;49(14):8804–14. 10.1021/acs.est.5b02641 26066997

[14] Li J, Gramatica P. Classification and virtual screening of androgen receptor antagonists. Journal of chemical information and modeling. 2010;50(5):861–74. 10.1021/ci100078u 20405856

[15] Kleinstreuer NC, Ceger P, Watt ED, Martin M, Houck K, Browne P, et al Development and validation of a computational model for androgen receptor activity. Chemical research in toxicology. 2016;30(4):946–64. 10.1021/acs.chemrestox.6b00347 27933809 PMC5396026

[16] Chierici M, Giulini M, Bussola N, Jurman G, Furlanello C. Machine learning models for predicting endocrine disruption potential of environmental chemicals. Journal of Environmental Science and Health, Part C. 2018;36(4):237–51. 10.1080/10590501.2018.1537155 30628533

[17] Idakwo G, Luttrell J, Chen M, Hong H, Zhou Z, Gong P, et al A review on machine learning methods for in silico toxicity prediction. Journal of Environmental Science and Health, Part C. 2018;36(4):169–91. 10.1080/10590501.2018.1537118 30628866

[18] Szafran AT, Mancini MA. The myImageAnalysis project: a web-based application for high-content screening. Assay and drug development technologies. 2014;12(1):87–99. 10.1089/adt.2013.532 24547743 PMC3934667

[19] Mukherjee R, Onel M, Beykal B, Szafran AT, Stossi F, Mancini MA, et al Development of the Texas A&M Superfund Research Program Computational Platform for Data Integration, Visualization, and Analysis Computer Aided Chemical Engineering. 46: Elsevier; 2019 p. 967–72. 10.1016/B978-0-12-818634-3.50162-4 31612156 PMC6791821

[20] Enders CK. Applied missing data analysis: Guilford press; 2010.

[21] Onel M, Kieslich CA, Guzman YA, Floudas CA, Pistikopoulos EN. Big data approach to batch process monitoring: Simultaneous fault detection and diagnosis using nonlinear support vector machine-based feature selection. Computers & chemical engineering. 2018;115:46–63. 10.1016/j.compchemeng.2018.03.025 30386002 PMC6205516

[22] Onel M, Kieslich CA, Pistikopoulos EN. A nonlinear support vector machine-based feature selection approach for fault detection and diagnosis: Application to the Tennessee Eastman process. AIChE Journal. 2019;65(3):992–1005. 10.1002/aic.16497 32377021 PMC7202572

[23] Mukherjee R, Sengupta D, Sikdar SK. Parsimonious use of indicators for evaluating sustainability systems with multivariate statistical analyses. Clean Technologies and Environmental Policy. 2013;15(4):699–706.

[24] Mukherjee R. Selection of Sustainable Process and Essential Indicators for Decision Making Using Machine Learning Algorithms. Process Integration and Optimization for Sustainability. 2017;1(2):153–63.

[25] Rogers J, Gunn S. Identifying Feature Relevance Using a Random Forest In: Saunders C, Grobelnik M, Gunn S, Shawe-Taylor J, editors. Subspace, Latent Structure and Feature Selection SLSFS 2005; Berlin, Heidelberg: Springer Berlin Heidelberg; 2006 p. 173–84.

[26] Tarca AL, Carey VJ, Chen X-w, Romero R, Drăghici S. Machine learning and its applications to biology. PLoS computational biology. 2007;3(6). 10.1371/journal.pcbi.0030116 17604446 PMC1904382

[27] Beykal B, Boukouvala F, Floudas CA, Sorek N, Zalavadia H, Gildin E. Global optimization of grey-box computational systems using surrogate functions and application to highly constrained oil-field operations. Computers & Chemical Engineering. 2018;114:99–110.

[28] Needham CJ, Bradford JR, Bulpitt AJ, Westhead DR. A primer on learning in Bayesian networks for computational biology. PLoS computational biology. 2007;3(8). 10.1371/journal.pcbi.0030129 17784779 PMC1963499

[29] Ben-Hur A, Ong CS, Sonnenburg S, Schölkopf B, Rätsch G. Support vector machines and kernels for computational biology. PLoS computational biology. 2008;4(10). 10.1371/journal.pcbi.1000173 18974822 PMC2547983

[30] Onel M, Beykal B, Ferguson K, Chiu WA, McDonald TJ, Zhou L, et al Grouping of complex substances using analytical chemistry data: A framework for quantitative evaluation and visualization. PloS one. 2019;14(10). 10.1371/journal.pone.0223517 31600275 PMC6786635

[31] Onel M, Beykal B, Wang M, Grimm FA, Zhou L, Wright FA, et al Optimal chemical grouping and sorbent material design by data analysis, modeling and dimensionality reduction techniques Computer Aided Chemical Engineering. 43: Elsevier; 2018 p. 421–6. 10.1016/B978-0-444-64235-6.50076-0 30534632 PMC6284807

[32] Beykal B, Boukouvala F, Floudas CA, Pistikopoulos EN. Optimal design of energy systems using constrained grey-box multi-objective optimization. Computers & chemical engineering. 2018;116:488–502. 10.1016/j.compchemeng.2018.02.017 30546167 PMC6287910

[33] Breiman L. Random forests. Machine learning. 2001;45(1):5–32.

[34] Menze BH, Kelm BM, Masuch R, Himmelreich U, Bachert P, Petrich W, et al A comparison of random forest and its Gini importance with standard chemometric methods for the feature selection and classification of spectral data. BMC bioinformatics. 2009;10(1):213. 19591666 10.1186/1471-2105-10-213 PMC2724423

