# Supplementary material for: Classification of estrogenic compounds by coupling high content analysis and machine learning algorithms
Source: PLoS Comput Biol. 2020 Sep 24;16(9):e1008191. doi: 10.1371/journal.pcbi.1008191 (PMC7538107; doi:10.1371/journal.pcbi.1008191)
Supplement: S1 File — (DOCX) [file pcbi.1008191.s001.docx]

**Supplementary Material for “Classification of estrogenic compounds by coupling high content analysis and machine learning algorithms”**

Rajib Mukherjee^1,2¶^, Burcu Beykal^1,2¶^, Adam T. Szafran^3^, Melis Onel^1,2^, Fabio Stossi^3,4^, Maureen G. Mancini^3,4^, Dillon Lloyd^5^, Fred A. Wright^5^, Lan Zhou^6^, Michael A. Mancini^3,4,7,8^, Efstratios N. Pistikopoulos^1,2*^

^1^ Artie McFerrin Department of Chemical Engineering, Texas A&M University, College Station, TX, United States of America

^2^ Texas A&M Energy Institute, Texas A&M University, College Station, TX, United States of America

^3^ Molecular and Cellular Biology, Baylor College of Medicine, Houston, TX, United States of America

^4^ GCC Center for Advanced Microscopy and Image Informatics, Houston, TX, United States of America

^5^ Bioinformatics Research Center, Center for Human Health and the Environment, Department of Statistics, North Carolina State University, Raleigh, NC, United States of America

^6^ Department of Statistics, Texas A&M University, College Station, TX, United States of America

^7^ Texas A&M University Institute for Bioscience and Technology, Houston, TX, United States of America

^8^ Pharmacology and Chemical Genomics, Baylor College of Medicine, Houston, TX, United States of America

* Corresponding author

Email: [stratos@tamu.edu](mailto:stratos@tamu.edu) (ENP)

^¶^ These authors contributed equally to this work.

The R Markdown documentation of the computational methodology is provided at: <http://parametric.tamu.edu/research/Mukherjee_etAl_2020_Rmarkdown.html>

High throughput microscopy and high content analysis-based experimental data can be downloaded at: <http://paroc.tamu.edu/Software/Mukherjee_etAl_2020_data.zip>
