# Supplementary material for: Classification of estrogenic compounds by coupling high content analysis and machine learning algorithms
Source: PLoS Comput Biol. 2020 Sep 24;16(9):e1008191. doi: 10.1371/journal.pcbi.1008191 (PMC7538107; doi:10.1371/journal.pcbi.1008191)
Supplement: S2 Fig — The ROCs for the “Array Area,” “Array PI Variance,” and “Array to Nucleoplasm Intensity Ratio” perfectly overlap and represent a perfect classifier in the training phase with AUC = 1. (DOCX) [file pcbi.1008191.s003.docx]

**S2 Figure. The training receiver operating characteristic (ROC) curves and the area under the curve (AUC) values for the 5 predictors.** The ROCs for the “Array Area,” “Array PI Variance,” and “Array to Nucleoplasm Intensity Ratio” perfectly overlap and represent a perfect classifier in the training phase with AUC = 1.
