# Supplementary material for: Classification of estrogenic compounds by coupling high content analysis and machine learning algorithms
Source: PLoS Comput Biol. 2020 Sep 24;16(9):e1008191. doi: 10.1371/journal.pcbi.1008191 (PMC7538107; doi:10.1371/journal.pcbi.1008191)
Supplement: S3 Fig — The percentage of variance explained by each component is provided in the axis labels in parenthesis. The cumulative variance explained by these two components is 95.47%. (DOCX) [file pcbi.1008191.s004.docx]

**S3 Figure. Visualization of the technical replicates of agonist and antagonist compounds using Principal component analysis (PCA).** The percentage of variance explained by each component is provided in the axis labels in parenthesis. The cumulative variance explained by these two components is 95.47%.
