# Supplementary material for: Classification of estrogenic compounds by coupling high content analysis and machine learning algorithms
Source: PLoS Comput Biol. 2020 Sep 24;16(9):e1008191. doi: 10.1371/journal.pcbi.1008191 (PMC7538107; doi:10.1371/journal.pcbi.1008191)
Supplement: S1 Table — (DOCX) [file pcbi.1008191.s005.docx]

**S1 Table. Logistic regression model validation results with all technical replicates of 32 active compounds for 17 biologically independent experiments with “Array PI Variance” as the model predictor.**

| Experimental Replicate | Accuracy | 95% CI | Sensitivity | Specificity | Balanced Accuracy |
| --- | --- | --- | --- | --- | --- |
| 1 | 0.84 | (0.77, 0.90) | 0.82 | 1.00 | 0.91 |
| 2 | 0.93 | (0.87, 0.97) | 0.97 | 0.62 | 0.80 |
| 3 | 0.80 | (0.72, 0.86) | 0.77 | 1.00 | 0.88 |
| 4 | 0.94 | (0.88, 0.97) | 0.98 | 0.62 | 0.80 |
| 5 | 0.95 | (0.89, 0.98) | 0.97 | 0.75 | 0.86 |
| 6 | 0.91 | (0.85, 0.96) | 0.90 | 1.00 | 0.95 |
| 7 | 0.92 | (0.86, 0.96) | 0.97 | 0.56 | 0.77 |
| 8 | 0.80 | (0.72, 0.86) | 0.77 | 1.00 | 0.88 |
| 9 | 0.94 | (0.88, 0.97) | 0.98 | 0.62 | 0.80 |
| 10 | 0.91 | (0.84, 0.95) | 0.90 | 0.94 | 0.92 |
| 11 | 0.89 | (0.82, 0.94) | 0.91 | 0.75 | 0.83 |
| 12 | 0.82 | (0.74, 0.88) | 0.79 | 1.00 | 0.90 |
| 13 | 0.93 | (0.87, 0.97) | 0.97 | 0.62 | 0.80 |
| 14 | 0.82 | (0.74, 0.88) | 0.79 | 1.00 | 0.90 |
| 15 | 0.93 | (0.87, 0.97) | 0.97 | 0.62 | 0.80 |
| 16 | 0.91 | (0.85, 0.96) | 0.92 | 0.88 | 0.90 |
| 17 | 0.97 | (0.92, 0.99) | 1.00 | 0.75 | 0.88 |
| Average | **0.89** | **-** | **0.90** | **0.81** | **0.86** |
