# Supplementary material for: Classification of estrogenic compounds by coupling high content analysis and machine learning algorithms
Source: PLoS Comput Biol. 2020 Sep 24;16(9):e1008191. doi: 10.1371/journal.pcbi.1008191 (PMC7538107; doi:10.1371/journal.pcbi.1008191)
Supplement: S2 Table — (DOCX) [file pcbi.1008191.s006.docx]

**S2 Table. Logistic regression model validation results with all technical replicates of 32 active compounds for 17 biologically independent experiments with “Array to Nucleoplasm Intensity Ratio” as the model predictor.**

| Experimental Replicate | Accuracy | 95% CI | Sensitivity | Specificity | Balanced Accuracy |
| --- | --- | --- | --- | --- | --- |
| 1 | 0.92 | (0.86, 0.96) | 0.91 | 1.00 | 0.96 |
| 2 | 0.88 | (0.80, 0.93) | 1.00 | 0.00 | 0.50 |
| 3 | 0.94 | (0.88, 0.97) | 0.93 | 1.00 | 0.96 |
| 4 | 0.88 | (0.80, 0.93) | 1.00 | 0.00 | 0.50 |
| 5 | 0.95 | (0.89, 0.98) | 1.00 | 0.56 | 0.78 |
| 6 | 0.93 | (0.87, 0.97) | 0.92 | 1.00 | 0.96 |
| 7 | 0.88 | (0.80, 0.93) | 1.00 | 0.00 | 0.50 |
| 8 | 0.96 | (0.91, 0.99) | 0.97 | 0.88 | 0.92 |
| 9 | 0.88 | (0.80, 0.93) | 1.00 | 0.00 | 0.50 |
| 10 | 0.97 | (0.92, 0.99) | 0.97 | 0.94 | 0.96 |
| 11 | 0.95 | (0.90, 0.98) | 1.00 | 0.62 | 0.81 |
| 12 | 0.98 | (0.93, 1.00) | 0.99 | 0.88 | 0.93 |
| 13 | 0.88 | (0.80, 0.93) | 1.00 | 0.00 | 0.50 |
| 14 | 0.94 | (0.88, 0.97) | 0.94 | 0.94 | 0.94 |
| 15 | 0.89 | (0.82, 0.94) | 1.00 | 0.12 | 0.56 |
| 16 | 0.96 | (0.91, 0.99) | 0.96 | 0.94 | 0.95 |
| 17 | 0.91 | (0.85, 0.96) | 1.00 | 0.31 | 0.66 |
| Average | **0.92** | **-** | **0.98** | **0.54** | **0.76** |
